# Production of a Blue Pigment (Glaukothalin) by Marine *Rheinheimera* spp.

**DOI:** 10.1155/2009/701735

**Published:** 2009-06-14

**Authors:** Hans-Peter Grossart, Marc Thorwest, Inken Plitzko, Thorsten Brinkhoff, Meinhard Simon, Axel Zeeck

**Affiliations:** ^1^Department of Limnology of Stratified Lakes, Leibniz-Institute of Freshwater Ecology and Inland Fisheries, Alte Fischerhuette 2, 16775 Neuglobsow, Germany; ^2^Institute of Organic and Biomolecular Chemistry, University of Göttingen, Tammannstrasse 2, 37077 Göttingen, Germany

## Abstract

Two *γ*-*Proteobacteria* strains, that is, HP1 and HP9, which both produce a diffusible deep blue pigment, were isolated from the German Wadden Sea and from the Øresund, Denmark, respectively. Both strains affiliate with the genus *Rheinheimera*. Small amounts of the pigment could be extracted from HP1 grown in a 50 L fermenter and were purified chromatographically. Chemical analysis of the pigment including NMR and mass spectrometry led to a molecular formula of C_34_H_56_N_4_O_4_ (m.w. 584.85) which has not yet been reported in literature. The molecule is highly symmetrically and consists of two heterocyclic halves to which aliphatic side chains are attached. The pigment has been named glaukothalin due to its blue color and its marine origin (*glaukos*, gr. = blue, *thalatta*, gr. = sea). Production of glaukothalin on MB2216 agar plates by our *Rheinheimera* strains is affected in the presence of other bacterial strains either increasing or decreasing pigment production. The addition of a single amino acid, arginine (5 gl^−1^), greatly increases pigment production by our *Rheinheimera* strains. Even though the production of glaukothalin leads to inhibitory activity against three bacterial strains from marine particles, our *Rheinheimera* isolates are inhibited by various bacteria of different phylogenetic groups. The ecological role of glaukothalin production by *Rheinheimera* strains, however, remains largely unknown.

## 1. Introduction

The genus *Rheinheimera* has been introduced by Brettar et al. [1] with *Rheinheimera baltica* as type species and further described species, such as *R. pacifica *[2] and *R. perlucida *[3], as well as several strains. These aerobic, chemoheterotrophic bacteria belong to the *γ*-*Proteobacteria* and have been isolated from various environments, such as the upper water layer of the Baltic Sea and deep sea water of the Pacific, and have been even found in Lake Kinneret [4]. The most obvious feature of *Rheinheimera baltica* is the production of a deep-blue and unpolar pigment of a so far unidentified chemical composition. The closest relative to the genus *Rheinheimera* is *Alishewanella fetalis*; however, physiology, fatty acids, and also color of this bacterium are very different [1, 2]. By 16S rRNA gene sequence analysis of various strains, Brettar et al. [1] demonstrated that *Rheinheimera baltica* is closely related to strains from other environments, including the deep-sea. The isolates were able to grow at a temperature range from 4 to 30°C and at salinities ranging from 0 to 30‰ indicating their high potential to adapt to changing environmental conditions.

Pigmentation is a common feature of bacteria of different phylogenetic and environmental origins There are several groups of bacterial pigments which are in general noncovalently bound to proteins. (a) Pigment-protein complexes are organized as photosynthetic units and consist either as photosynthetic reaction centers or as light-harvesting complexes. Pigmentation of purple bacteria has been studied extensively [5]. (b) Phenanzine pigments [6, 7] are known from several bacterial genera in more than 50 varieties, each of which contains a substituted phenazine ring system, and together they represent every color of the visible spectrum. Phenazines are derived from the shikimic acid pathway via phenazine-1,6-dicarboxylic acid and seem to be precursors for further metabolism or are used as redox systems. (c) Other bacterial pigments such as carotenoids protect the organism from ionizing radiation. Ionizing radiation produces electrons, hydroxyl radicals, and hydride radicals which are capable of altering biopolymers, for example, proteins and DNA. Higher pigmentation of bacteria due to increased UV radiation has been reported for bacteria in surface waters [8]. (d) In addition, violacein, a pigment with putative antibiotic and/or antiviral activity, has been shown to even influence protozoan grazing [9].

The production of the intensive blue pigment is one of the most striking features of the *Rheinheimera baltica* group, but yet nothing is known about its chemistry, production dynamics, and ecological role. There are many recent indications that production of pigments greatly depends on environmental conditions including interactions with other bacteria in the surrounding environment [10]. Angell et al. [11] could demonstrate that production of a blue pigment with antibiotic activity (pyocyanin) by *Pseudomonas aeruginosa *was induced when kept in a coculture with an *Enterobacter* species (Pup14B). Some dual microbial systems have been characterized on the molecular level, and several small signaling molecules are known. Therefore, our study aims at studying environmental parameters such as growth medium and interspecific interactions for optimum pigment production.

## 2. Material and Methods

### 2.1. Isolation

Two blue pigmented *γ*-*Proteobacteria* strains (HP1 and HP9 of the genus *Rheinheimera*) were isolated in June 1999 from (a) diatom aggregates (mainly composed of *Skeletonema costatum, Chaetoceros sp*., and *Coscinodiscus sp*.) of Øresund in Helsingør, Denmark and (b) from organic particles in the German Wadden Sea. The isolates were grown on agar plates (1%-2 % w/w) enriched with (0.1%–2% w/w) Marine Broth (MB2216, Difco, USA) at *in situ* temperature (15°C) in the dark. Single colonies were transferred at least five times until considered as pure. Purity was checked by colony morphology and color and by denaturing gradient gel electrophoresis (DGGE) of PCR-generated 16S rRNA gene fragments [12]. Isolates were considered as pure when showing a single DGGE band and were sequenced thereafter.

### 2.2. Sequencing and Phylogeny

Chromosomal DNA was extracted and sequenced as described by Grossart et al., 2004 [13]. Sequences were compared with similar sequences of reference organisms by BLAST search (http://www.ncbi.nlm.nih.gov/blast). Phylogenetic reconstructions were done using the ARB software package (http://arb-home.de/) [14]. For tree calculation, 16S rRNA gene sequences were aligned automatically using the integrated alignment module within the ARB package and subsequently corrected manually. Validity of branching patterns of the trees was checked by applying 3 phylogenetic reconstruction methods: neighbor-joining, maximum parsimony, and maximum likelihood to the appropriate sets of sequences. Alignment positions at which less than 50% of sequences of the entire set of data had the same residues were excluded from the calculations to prevent uncertain alignments within highly variable positions of the 16S rRNA genes, which cause mistakes in tree topology.

The nucleotide sequences of the isolates sequenced in this study are available from GenBank under accession number. AY241547 (HP1) and AY359588 (HP9).

### 2.3. Communication Screening

**Figure 1:**
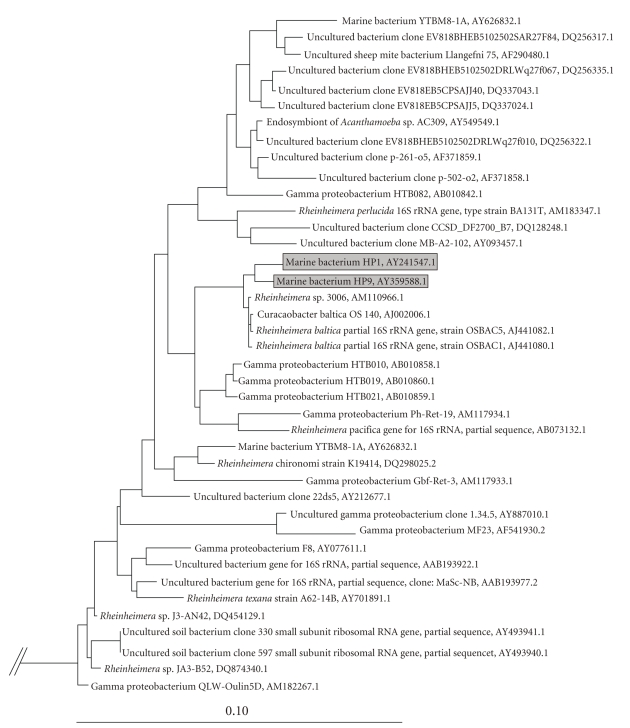
Neighbor-joining tree showing the phylogenetic affiliation of strains HP1 and HP9. Selected sequences from the *α* subclass of *Proteobacteria* were used to root the tree. The bar indicates 10% sequence divergence.

To test whether production of the blue pigment by HP1 and HP9 is different in the presence of other strains, we have introduced communication screening. The isolates were plated in an alternate pattern with 18 different isolates of various phylogenetic origin (Table 1, Figure 1). All tests were performed on Marine Broth agar plates (1%-2% w/w, 37 gl^−1^ MB2216, Difco, USA) at in situ temperature (15°C) in the dark. We performed several inhibition tests according to Grossart et al. (2004) [13]. The assay discs were transferred onto freshly prepared bacterial lawns (50 *μ*L of isolate suspension ca. 10^8^ cells mL^−1^) of 18 different bacterial strains (Table 1). The assays were screened for formation of inhibition zones every day for up to 20 days of incubation. We have also studied the biological activity of glaukothalin in standard cytotoxic tests [15] against higher organisms such as crustaceans (*Artemia salina*) and nematodes (*Caenorhabditis elegans*).

### 2.4. Effect of Growth Media

Nutrient rich or poor media prove whether pigment formation depends on growth media. Marine Broth (MB2216, Difco, USA) is rich in amino acids and proteins whereas artificial seawater (ASW) [16] only contains traces of organic matter. Cells of HP1 and HP9 were washed in sterile ASW (centrifugation at 1000 rpm) at least three times before incubation. Both strains were also grown on LB (Fluka 61748) agar plates (1%-2%) at 5‰ salinities either without or with addition of a single amino acid (arginine or glycine, each 5 gL^−1^) known to stimulate bacterial pigment production (A. Zeeck, unpublished data). To test for the effect of salinity, both strains were grown on LB agar plates at either 5‰ or 25‰ salinity.

### 2.5. Chemical Analysis

Strain HP1 was cultivated in a 50-liter fermenter with MB2216 medium. Inoculation of the fermenter was done with 4 L diluted seed culture (10%), and the incubation occurred at 28°C for ca. 7 days under continuous aeration (0.66 air:medium, v/v). The agitation was maintained at 200 rpm until a cell density of 10^11^ to 10^12^ cells L^−1^ was reached. The resulting culture broth was filtered cell-free and extracted three times with 15 L of ethyl acetate using an Ultra-Turrax to homogenize the organic and aqueous layers. The combined organic layers were concentrated by vacuum yielding 4.1 g of an oily residue. Thin-layer chromatography (TLC) of the crude extract on silica gel (60 F_254_ on aluminum or glass plates, 0.25 mm layer, Merck) with CHCl_3_ as a solvent indicated a blue spot with an *R*_f_-value of 0.34. To obtain the pure pigment, the crude extract was chromatographed on a silica gel column using a gradient of CHCl_3_/MeOH (1 : 0 to 7 : 1) and purified on Sephadex LH-20 (CHCl_3_) and Sephadex LH-20 (CH_2_Cl_2_), yielding 3 mg (<0.1 mg/L) in total.

The infrared spectrum of the purified pigment was recorded with a Perkin-Elmer FT IR-1600 (KBr disc) spectrometer and the UV spectra on a Varian Cary 3E spectrophotometer. Chemical analyses comprised Atomic Adsorption Spectroscopy (*AAS)*, mass spectrometry including *DCI-MS* and *HRESI-MS*, and nuclear magnetic resonance methods such as *^1^H-NMR, ^13^C-NMR, ^1^H,^1^H-COSY, HMBC*, and *HSQC. *Details of the chemical analyses performed are described elsewhere Thorwest [17].

## 3. Results and Discussion

### 3.1. Isolation and Phylogeny

We have isolated one blue-colored strain (HP9) from natural diatom aggregates (mainly composed of *Skeletonema costatum, Chaetoceros *sp., and *Coscinodiscus *sp.) of Øresund in Helsingør, Denmark, in June 1999. The second blue-colored strain (HP1) has been isolated from macroscopic organic particles of the German Wadden Sea. Phylogenetic analysis revealed that both of our isolates are closely related to each other and with organisms of the genus *Rheinheimera * (Figure 1) [1]. Organisms of this cluster appear to be widely distributed since they were obtained from marine, limnetic, and terrestrial habitats, like the Baltic Sea, the North Sea, the Pacific Ocean, Lake Kinneret, Chesapeake Bay, the river Weser estuary, river snow of the South Saskatchewan river, and Swiss chard rhizoplane. The cluster also contained isolates from the deep sea (HTB010, HTB019, HTB021) [18]. Unfortunately, these authors cannot give any information on pigmentation of their deep-sea strains (H. Takami, pers. communication). Another isolate (SELECT1) also has close affiliation with the *Rheinheimera* cluster (see “BLAST distance tree of results”) and produces a violet pigment [19] which may be identical to our *Rheinheimera* strains.

### 3.2. Chemical Characterization of the Pigment

**Figure 2:**
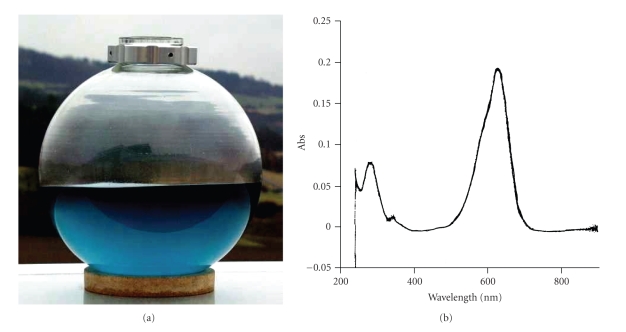
*Glaukothalin* isolated from *Rheinheimera *sp. strain HP1 grown in a 50 L fermenter on Marine Broth (MB2216) medium: (a) blue color, (b) UV spectrum in CHCl_3_.

The extraction of 40 L culture broth of HP1 resulted in 3.0 mg of pure pigment. Due to its blue color and marine origin we named this compound glaukothalin (*glaukos*, gr. = blue, *thalatta*, gr. = sea, Figure 2(a)). Glaukothalin is readily soluble in pyridine or HMPT, moderately soluble in DMSO, DMF or CHCl_3_, and insoluble in acetone, methanol, water (acidic or alkaline), benzene or cyclohexane. The UV spectrum of glaukothalin in CHCl_3 _(Figure 2(b)) exhibits characteristic absorption maxima at 636 nm (log *ε* = 4.51), 582 nm (sh), 286 nm (sh), 241 nm. Addition of acetic acid resulted in a small bathochromic shift (639 nm, 582 nm (sh), 279 nm (sh), 241 nm). In the presence of diethylamine the spectrum remains unchanged. The intensive blue color and the unique solubility of glaukothalin suggested the presence of a metal complex, but AAS did not confirm the presence of any metal atom. DCI-MS and HRESI-MS (M^+^: m/z 584.4320) have been used to establish the molecular formula of glaukothalin as C_34_H_56_N_4_O_4_ (m.w. 584.85). Natural products with this molecular formula have not been described in the literature so far. The molecule is highly symmetric (C_17_H_28_N_2_O_2_)_2_ and consists of two conjugated ring structures to which aliphatic side chains are attached. Each ring system consists of five carbon atoms (sp^2^-hybridized), one NH-group (*δ*_*H*_ = 13.8), and two oxygen atoms. Only one of the carbon atoms (*δ*_*H*_ = 105.7) carries a hydrogen atom (*δ*_*H*_ = 8.82). The remaining quarternary carbon atoms generate the following ^13^C –NMR signals: *δ*_*C*_ = 125.6, 137.2, 161.8, 167.1. Additional information on chemical characterization and structure elucidation of glaukothalin is given in the supplemental and will be reported in a subsequent study [20]. The ring system is smaller and differs from that of phytochromobilin, which has been isolated from algae and cyanobacteria [21, 22]. The chemical structure of glaukothalin is different from that of other known bacteriochromophores; thus a possible role as phytochrome-type photoreceptor seems to be unlikely.

### 3.3. Factors Regulating Pigment Production

**Figure 3:**
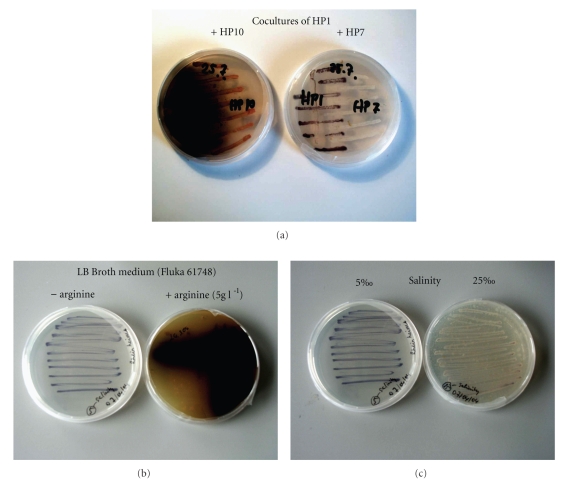
Production of the *glaukothalin* by *Rheinheimera *sp. strain HP1 (a) grown on Marine Broth (MB2216) medium in the presence of HP10 or HP7 (note the intense production in the presence of HP10), (b) grown on LB Broth medium (Fluka 61748) at 5‰ salinities either without or with the amino acid arginine (5 g L^−1^), (c) grown on LB Broth medium at salinities of 5‰ and 25‰.

Our screening assays revealed that growth and pigment production by HP1 and HP9 differs in the presence of other strains (Figure 3(a), Table 1). Both isolates behaved very similarly in terms of pigment production when other isolates were present. This suggests that production of glaukothalin by both strains depends on the presence of specific chemical substances such as signaling molecules, for example, acylated homoserine lactones (AHL) and amino acids (see below). It has been shown that production of pigments, for example, pyocyanin [11], is under quorum control and affected by the presence of other bacterial strains [10]. Gram et al. [23] demonstrated that bacteria isolated from marine snow and marine diatoms appear to be capable of producing AHLs. However, strains HP1 and HP9 did not produce any AHL, and pigment production remained unchanged in the presence of *N*-3-oxo-hexanoyl-homoserine lactone (OHHL), *N*-hexanoyl-homoserine lactone (HHL), and *N*-octanoyl-homoserine lactone (OHL), all from Sigma Chemicals. Hence, we do not have any indication that AHL standards induce glaukothalin production even though release of glaukothalin into the surrounding medium occurs at high cell densities of HP1 and HP9 (>10^11^ l^−1^).

In addition, production of glaukothalin by strains HP1 and HP9 always occurred when growing on MB2216 agar medium. It was much lower or even absent when the isolates were growing on LB agar or in artificial seawater medium (ASW, with only traces of labile organic matter), respectively. This result suggests that the availability of soluble organic matter affects pigment production by both strains. To test whether glaukothalin production can be stimulated by the addition of small molecules such as specific amino acids, we have added arginine or glycine to LB agar plates on which pigmentation of both strains was moderate (see Figure 3(b)). While the addition of glycine did not have any effect on pigment production by HP1 and HP9, addition of arginine greatly increased the production of glaukothalin (Figure 3(b)). Recent studies [11, 24] showed that the presence of specific organics can be crucial for microbial fermentation and even pigmentation. Arginine is an N-rich amino acid and is not produced by all bacteria and is well known to be important for specific adaptations of bacteria to their specific environment.

Another environmental parameter which negatively affected pigment production is salinity (Figure 3(c)). Hence, a combination of low salinities and addition of arginine to our *Rheinheimera* strains led to greatly increase production of glaukothalin*.* This has great implications for optimizing production of this new pigment on a larger scale.

### 3.4. Potential Ecological Role of Glaukothalin

Our inhibition tests showed that strains HP1 and HP9 were able to inhibit three isolates (two isolates of the *Cytophaga Flavobacter Bacteroides* group: strains HP27 and 28 and one isolate of the *Bacillus/Clostridium* group: strain HP10 [13]. On the contrary, growth of strains HP1 and HP9 was inhibited by a variety of strains from different bacterial subgroups (Table 1). Thus, inhibition patterns are specific to individual strains (Table 1) with various phylogenetic origin [13]. It has been shown that pigments, for example, phenanzines [7], exhibit antibiotic activities. Phenazines have several functions such as redox cycling and generation of toxic oxygen radicals which probably account for their often observed antibiotic activity. The ecological importance of phenazine production appears to be related to their broad spectrum of antibiotic activity and their ability to act as in vivo virulence factors [7].

Therefore, we have also studied the biological activity of glaukothalin in cytotoxic tests against higher organisms such as crustaceans (*Artemia salina*) and nematodes (*Caenorhabditis elegans*). The tests showed that glaukothalin is inactive against *Caenorhabditis elegans*, but that it exhibits a strong biological activity against *Artemia salina* (*c* = 0.1 mg/mL, mortality = 100%). These results suggest that production of glaukothalin is linked to antibiotic activity as well as to cytotoxicity.

The core structure of glaukothalin is different to that of the pigment phycocyanin [11] and of phytochromobilins [26]. Hence, it remains questionable whether glaukothalin plays an analogous role for *Rheinheimera* strains. Further studies are needed to elucidate the ecological role of this new and unique pigment.

## Figures and Tables

**Table 1:** Growth inhibition and influence on pigment production of HP1 and HP9 by other strains during cocultivation on agar plates (see Figure 3(a)).

Phylum or group	Strain	Identification by GenBank alignment	%Homology to GeneBank sequence	Family	HP1/HP9	
					Inhibition	Pigmentation (visible on agar plates)
*α*-proteobacteria	(1) HP12	AF359535, *Roseobacter* strain ATAM407	98	Rhodobacteraceae		Violet
	(2) HP13	AF359546, marine bacterium SCRIPPS 739	96	Rhodobacteraceae		
	(3) HP29w	AF098495, Roseobacter strain ISM (uncultured)	94	Rhodobacteraceae	++	
	(4) HP33	AF345550, *Rhizobium sp.* Strain SDW052	99	Rhizobraceae		
		AF388033, *A tumef.aciens*	99			
	(5) T5	AJ296158, *Ruegeria* strain PP-154	99	Rhodobacteraceae		

*γ*-Proteobacteria	(6) HP3	AF062642, *Alcanivorax bork umensis*	98	Halomonadaceae	+	Blue
	(7) HP6	AJ000647, *Marinobacter * strain PCOB-2	99	Alteromonadacae	+	

CFB^(a) ^	(8) HP2	AF235114, *Cytophaga* strain KTO2ds22	98	Flexibacteraceae		Dark-violet
	(9) HP11	M58792, *Microscilla furvescens*	90	Flexibacteraceae		Violet
	(10) HP14	AF235114, *Cytophaga* strain KTO2ds22	98	Flexibacteraceae		Brown
	(11) HP25	AF277514, *Cellulophaga* strain SIC834 (uncultured)	98	Flavobacteriaceae	+	Brown
	(12) HP35	AF235114, *Cytophaga* strain KTO2ds22	98	Flexibacteraceae		Blue
	(13) HP44	AF235114, *Cytophaga* strain KTO2ds22	98	Flexibacteraceae		Brown

Actinomycetes	(14) HP5	AF321022, *Frigobacterium* strain GOB	98	Microbacteriaceae	+	Blue
	(15) HP7	AF197036, *Arthrobacter* strain SMCC G980	97	Micrococcaceae	+	Violet

Bacillus-Clostridium	(16) HP8	AY038905, marine bacterium SE165	97	Bacillaceae		Brown
	(17) HP9w	AF156315, marine *bacillus* strain NRRLB-14904	98	Bacillaceae	++	Brown
	(18) HP10	AF275714, *Haeler* soda lake bacterium Z6	99	Bacillaceae		Brown

^(a) ^CFB, Cytophaga-Flavobacterium-Bacteroides, ++ (inhibition zone >5 mm), + (inhibition zone ≤5 mm).
